# A single nucleotide polymorphism in the *Plasmodium falciparum atg18* gene associates with artemisinin resistance and confers enhanced parasite survival under nutrient deprivation

**DOI:** 10.1186/s12936-018-2532-x

**Published:** 2018-10-26

**Authors:** Kimberly F. Breglio, Roberto Amato, Richard Eastman, Pharath Lim, Juliana M. Sa, Rajarshi Guha, Sundar Ganesan, David W. Dorward, Carleen Klumpp-Thomas, Crystal McKnight, Rick M. Fairhurst, David Roberts, Craig Thomas, Anna Katharina Simon

**Affiliations:** 1 National Center for Advancing Translational Sciences, National Institutes of Health, Bethesda, MD USA; 2 Nuffield Department of Medicine, University of Oxford, Oxford, UK; 3 Wellcome Sanger Institute, Wellcome Genome Campus, Hinxton, Cambridge, UK; 4 National Institute of Allergy and Infectious Diseases, National Institutes of Health, Bethesda, MD USA; 5 Vertex Pharmaceuticals, Boston, MA USA; 6 Rocky Mountain Laboratories, National Institute of Allergy and Infectious Diseases, National Institutes of Health, Bethesda, MD USA; 7 Radcliffe Department of Medicine, Medical Sciences Division, University of Oxford, Oxford, UK; 8 Kennedy Institute of Rheumatology and Medical Research Council Human Immunology Unit, Weatherall Institute of Molecular Medicine, University of Oxford, Oxford, UK

**Keywords:** Artemisinin resistance, Autophagy, Fitness, Drug resistance, atg18

## Abstract

**Background:**

Artemisinin-resistant *Plasmodium falciparum* has been reported throughout the Greater Mekong subregion and threatens to disrupt current malaria control efforts worldwide. Polymorphisms in *kelch13* have been associated with clinical and in vitro resistance phenotypes; however, several studies suggest that the genetic determinants of resistance may involve multiple genes. Current proposed mechanisms of resistance conferred by polymorphisms in *kelch13* hint at a connection to an autophagy-like pathway in *P. falciparum*.

**Results:**

A SNP in *autophagy*-*related gene 18* (*atg18*) was associated with long parasite clearance half-life in patients following artemisinin-based combination therapy. This gene encodes PfAtg18, which is shown to be similar to the mammalian/yeast homologue WIPI/Atg18 in terms of structure, binding abilities, and ability to form puncta in response to stress. To investigate the contribution of this polymorphism, the *atg18* gene was edited using CRISPR/Cas9 to introduce a T38I mutation into a *k13*-edited Dd2 parasite. The presence of this SNP confers a fitness advantage by enabling parasites to grow faster in nutrient-limited settings. The mutant and parent parasites were screened against drug libraries of 6349 unique compounds. While the SNP did not modulate the parasite’s susceptibility to any of the anti-malarial compounds using a 72-h drug pulse, it did alter the parasite’s susceptibility to 227 other compounds.

**Conclusions:**

These results suggest that the *atg18* T38I polymorphism may provide additional resistance against artemisinin derivatives, but not partner drugs, even in the absence of *kelch13* mutations, and may also be important in parasite survival during nutrient deprivation.

**Electronic supplementary material:**

The online version of this article (10.1186/s12936-018-2532-x) contains supplementary material, which is available to authorized users.

## Background

Artemisinin (ART)-resistant *Plasmodium falciparum* has been reported in the Greater Mekong Subregion since 2007. Artemisinin-based combination therapy (ACT), which pairs a short-acting ART derivative with a long-acting partner drug, is the mainstay of anti-malarial treatment and is likely to have been partly responsible for significantly decreasing malaria-related morbidity and mortality over the past 15 years. The slow parasite clearance rates following ACT suggest resistance to ART derivatives. This resistance places increasing selective pressure for variants or traits that confer resistance to ACT partner drugs and has led to the rapid failure of several artemisinin-based combinations, including dihydroartemisinin-piperaquine in Cambodia [1, 2]. Single nucleotide polymorphisms (SNPs) in *kelch13* (*k13*) have been associated with slow parasite clearance rates in response to ACT [3] and increased in vitro parasite survival in response to ART derivatives [4, 5].

In clinical isolates, *k13* polymorphisms appear on a genetic background comprising polymorphisms in *apicoplast ribosomal protein S10* (*arps10*), *chloroquine resistance transporter* (*crt*), *ferrodoxin* (*fd*), and *multidrug*-*resistance protein 2* (*mdr2*) [6]. Although parasites with *k13* mutations have been found elsewhere in the world, including Africa, they are not always associated with long parasite clearance half-life in response to ACT [7–12]. Therefore, the genetic background specific to some areas in Southeast Asia may be responsible for some of the drug resistance phenotype. Alternatively, these background mutations may be important in transmission or confer a survival advantage over parasites without this array of SNPs. Indeed, overcoming the cellular stress response following ART treatment may underlie the parasite resistance mechanism. Autophagy is one such cellular stress response that may be employed by a parasite and therefore may not only be modified by genetic variants that promote survival but also may represent a potential target pathway for novel anti-malarial compounds.

Autophagy, an intracellular process that degrades and recycles damaged organelles, has been well-characterized in many organisms, but is not well-described in *P. falciparum*. A limited set of putative autophagy-related proteins is encoded by the *P. falciparum* genome, but the functions of most of these proteins have yet to be defined. Two of these autophagy-related proteins, autophagy-related protein 8 (PfAtg8) and autophagy-related protein 18 (PfAtg18), have been shown to co-localize with the apicoplast and to be involved in apicoplast inheritance [13]. Homologous Atg8 and Atg18 proteins in yeast and mammals have been shown to form puncta during the upregulation of autophagy [14]. Autophagy was investigated as a possible mechanism related to ART resistance due to several connections between an autophagy-like pathway in *P. falciparum* and known mechanisms of ART action and resistance [15]. Indeed, ART can damage cells by reactive oxygen species (ROS) [16] and ROS are potent activators of autophagy [17]. This would indicate that ARTs, even in the absence of resistance, could induce an autophagy-like pathway. Several possible resistance mechanisms have been posited wherein the resistant isolate is able to withstand the deleterious effects of ART based on an ability to withstand oxidative stress. Another hint at a connection to autophagy is through increased levels of phosphatidylinositol 3-phosphate (PI3P), a lipid regulating autophagy, that occur in ART-resistant isolates [18]. Lastly, the upregulation of the unfolded protein response (UPR), a process that induces autophagy, is associated with resistance [19]. Therefore, an ostensible connection between an autophagy-like pathway and ART resistance in *P. falciparum* was investigated.

Several polymorphisms were found in autophagy-related genes that associate with drug-resistant phenotypes, most interestingly a T38I SNP in *autophagy*-*related gene 18* (*atg18*), which associates with long parasite clearance half-life following ACT. The protein encoded by this gene has similar structural domains, binding capabilities, and behaviour as mammalian and yeast homologues. A T38I CRISPR-edited line was created that determined that the SNP confers increased parasite survival in a nutrient-limited setting, as well as differential susceptibility to over 200 compounds in a 72-h assay that measures the half-maximal inhibitory concentration (IC_50_) values for ART derivatives.

## Methods

### Bioinformatics of autophagy-related genes

Putative autophagy-related genes in the *P. falciparum* genome were found through the conversion of a previously published list of human autophagy-related genes from Behrends et al. [20] and supplemented with autophagy-related genes appearing on the PlasmoDB and Malaria Parasite Metabolic Pathways (MPMP) websites.

A sub-analysis of a previously performed genome-wide association study (GWAS) on 782 isolates from Southeast Asia was performed to determine if any SNPs in genes involved in autophagy were associated with slow parasite clearance rates following ART treatment or partner drug resistance (IC_50_ values). The resistance phenotype was a quantitative trait in a linear mixed model. A Bonferroni correction was applied, placing statistical significance at p-values less than 5E−6.

IC_50_ experiments for chloroquine, piperaquine, quinine, artesunate, and DHA were performed ex vivo in Cambodia on a subset of isolates from the GWAS, namely those isolates from in the NIH Cambodian research sites, as previously described [21] and used a SYBR green fluorescence readout [22], assessed using a FLUOstar OPTIMA. IC_50_ calculations were performed using the online IVART IC50 analysis, Worldwide Antimalarial Resistance Network (http://www.wwarn.org/tools-resources/toolkit/analyse/ivart).

These data were pooled and matched with sequencing data from whole genome sequencing (WGS). Samples were excluded from the IC_50_ analyses if data were missing for either *atg18* (n = 265) or *k13* (n = 311) or both (n = 196) or if the final sequence was heterogeneous for that locus (*atg18*: n = 22; *k13*: n = 37). In total, 334 isolates were excluded, making the final cohort of 448 samples. Data were analysed using GraphPad Prism 6, unpaired *t* test.

### *Plasmodium falciparum* line production

The homology region sequences were designed to include the *atg18* SNP, changing the 38th amino acid from a threonine to an isoleucine (T38I). The protospacer adjacent motif (PAM) guide sequence was edited to include multiple synonymous SNPs to prevent cutting of the new sequence via the PAM-directed Cas9. PciI sites flank the sequences for later infusion reactions. The pL6 plasmid with human dihydrofolate reductase (*hDHFR*) was modified as described by Ghorbal and colleagues using In-Fusion cloning (Clontech) to include the desired single guide RNA (sgRNA) [4]. A DNA construct for the homology repair region was made by gene synthesis (GENEWIZ) and added to the pL6 plasmid via In-Fusion. sgRNA and homology region sequences were confirmed using DNA sequencing (GENEWIZ). The pUF1-Cas9 plasmid was used for Cas9 expression with yeast dihydroorotate dehydrogenase (*yDHODH*) resistance cassette [4].

The plasmid backbone for pDC2 AttP BSD was obtained from Dr. Richard Eastman at the National Center for Advancing Translational Sciences (NCATS). The sequence for *atg18* with an N-terminal green fluorescent protein (GFP) was made by Integrated DNA Technologies and inserted into pDC2 AttP plasmid with a BSD resistance cassette and ef1α promoter. Plasmid sequences were confirmed by PCR and DNA sequencing (GENEWIZ).

Uninfected erythrocytes were electroporated in the presence of plasmids using a Bio-Rad Gene Pulser Xcell and Percoll-isolated late-stage parasites were allowed to invade as previously described [23–25]. Selection for pUF1-Cas9 plasmids was with 1.5 μM DSM1, selection for pL6 was with 2 nM WR99210, and selection for pDC2 AttP BSD was with 4 μM blasticidin (BSD). Drug selection media was changed every day for 1 week until all parasites appeared dead by microscopy. Media was then changed three times a week and blood was added once weekly. Smears were checked three times a week for parasites until day 60 post-transfection. Parasites were detected by Giemsa-stained smears and were screened for editing by PCR. Cultures with positive PCR products associated with editing were sequenced, then cloned by limiting dilution and sequence verified (GENEWIZ). The *atg18* T38I locus of Dd2^R539T^, a laboratory line (Dd2) with a R539T Kelch13 mutation [5], was edited to create the mutant Dd2^R539T/T38I^.

### Drug screen

Isogenic parasite lines Dd2^R539T^ and Dd2^R539T/T38I^ were screened at NCATS against Mechanism Interrogation Plate (MIPE) 4.1 library [26] and NPACT library (https://ncats.nih.gov/preclinical/core/compound/npact). MIPE 4.1 contained 1978 compounds, which were screened against the lines at 11 concentrations. NPACT contained 5632 compounds, which were screened at seven concentrations. Because NPACT and MIPE contain some of the same compounds, the total screened were 6349 unique compounds. Concentrations screened were three-fold dilutions for MIPE from 29 µM to 0.5 nM, or five-fold dilutions for NPACT from 29 µM to 1.86 nM. Parasite lines were diluted to 0.3% parasitaemia at 4% Hct and dispensed using a Multidrop Combi in 5 µL volume and 3 µL complete media into 1536-well black clear cyclo-olefin polymer plate plates for a final hematocrit of 2.5%. Compounds were plated with 23 nL of each compound using a pin tool [27]. Plates were incubated for 72 h at 37 °C to allow for 1.5 generations of intraerythrocytic parasite growth [28].

Cells were lysed with SYBR Green I in lysis buffer and incubated overnight (approximately 18 h) in the dark [29, 30]. Fluorescence was measured using an EnVision plate reader at 485/14 nm excitation and 535/25 nm emission. The dose response data was fitted to the 4-parameter Hill equation using a grid-based algorithm developed in house [31]. For compounds with valid curve fits, an IC_50_ value was obtained. In addition, fits were classified into curve classes, a heuristic scheme that allows one to easily categorize fits as good quality (i.e., well defined upper and lower asymptotes, greater than 80% efficacy), inconclusive (missing an asymptote or displaying activity at the highest dose) or inactive (no dose response). Statistical analysis to identify statistically significant differences in the responses at the maximum dose (MaxR) was done using ANOVA followed by Tukey post hoc test. Determination of class enrichment was assessed using Fishers test with Benjamini–Hochberg correction for multiple comparisons.

### Atg18 alignment with homologues

Genes for proteins in the WIPI family were aligned using MegAlign version 12.2.0 using protein sequences from *Homo sapiens* WIPI1 (AAH39867.1), WIPI2a-d (NP_056425.1, NP_057087.2, NP_001028690.1, NP_00102891.1) and *P. falciparum* Atg18 (XP_001347411.1). Analysis was performed using the Clustal V method, although relevant findings did not differ when Clustal W or Jotun Hein methods were used.

Using the sequence from PlasmoDB to the currently available crystal structure of Hsv2 in *K. lactis*, a model of PfAtg18 was created with UCSF Chimera version 1.10.1. The sequence for PI3P binding, FRRG, was highlighted in addition to the SNP of interest, T38I, and Atg16 binding site. The phosphorylation site score of T38 was determined using NetPhos 3.1 server to predict the likelihood of the threonine being a phosphorylation site for one of 17 kinases [32].

### PIP binding assay

The ability of Dd2^R539T^ to bind to various lipids, including PI3P, was assessed using a PIP Strip (Echelon). A GFP-trap_M (Chromotek) was used to isolate GFP-tagged Atg18 from Dd2^R539T^ parasites with Atg18-GFP, according to the manufacturer’s instructions. The PIP Strip membrane was then prepared according to the manufacturer’s instructions using the isolated Atg18-GFP, then stained with anti-GFP antibody (Abcam, ab290) at 1:2000 for 1 h at room temperature. Anti-rabbit HRP secondary antibody was used at 1:2000 for 1 h at room temperature followed by incubation with K-TMBP (Echelon) for development for 10 min. The membrane was then imaged to detect binding. The resulting image was processed using ImageJ. Briefly, the image was converted to 32-bit grayscale, background was subtracted with a radius set to 25 pixels, and the image was inverted. A circular selection was created and the integrated density of the center of each dot on the membrane was measured. The value of the blank on the membrane was subtracted out and anything below the blank was recorded as zero.

### Confocal slide production

Dd2^R539T^ lines with GFP-tagged Atg18 were used for confocal imaging studies. Cultures were washed three times with PBS, then were fixed with 0.0075% glutaraldehyde, 2% paraformaldehyde in PBS at room temperature for 30 min, washed with PBS three times, and permeabilized with 0.1% Triton X-100 (Sigma) in PBS for 10 min at room temperature. Parasites were washed with PBS three times and then stained with anti-GFP rabbit polyclonal (Abcam ab290) primary antibody overnight at 4 °C, washed with PBS three times, then stained with goat polyclonal to rabbit secondary antibody (Abcam ab150079, AF647) for 2 h at room temperature. Cells were washed three times with PBS and slides were made using Prolong Gold mounting solution with 4′,6-diamidino-2-phenylindole (DAPI) (ThermoFisher). Coverslips were placed on top of each slide, slides were covered with tin foil, and left to dry overnight at room temperature. Slides were stored at 4 °C before imaging. Images were collected on a Leica SP5 inverted confocal microscope with a 63 × oil immersion objective NA 1.4 (Leica Microsystems, Buffalo Grove, IL). Post-processing and image analysis were performed using Huygens (SVI imaging, Nederland) and Imaris software (Bitplane Inc., South Windsor, CT).

### Electron microscopy

Parasite culture Dd2^R539T^ with GFP-labelled Atg18 were synchronized to trophozoite and schizont stages using a Percoll gradient separation and then exposed to 700 nM DHA for 10 min, 1 h, or left unexposed. Cultures were pelleted and washed with PBS then pelleted and resuspended in 4% paraformaldehyde and 0.1% glutaraldehyde. Samples were stored at 4 °C before being shipped to Rocky Mountain Laboratories for processing and imaging.

For transmission electron microscopy of immune-labelled sections, samples were prepared essentially as previously described [33] with several adaptations as follows. For embedment, LR White acrylic resin (Electron Microscopy Sciences, Hatfield, PA) was used rather than Araldite epoxy resin. Silver sections were mounted on nickel grids, then dried overnight at 45 °C. The treatments with formic acid and sodium meta-periodate were omitted. Before labeling, grids were immersed in 100% ethanol for 10 s, then quickly rinsed with deionized water, and placed into blocking buffer. Primary and secondary antibodies were used at 1:50 dilution in blocking buffer. A rabbit anti-eGFP polyclonal primary antibody (Thermofisher OSE00003G) was used overnight at 4 °C following microwave irradiation.

### Nutrient deprivation assays

The ability of parasite lines Dd2, Dd2^R539T^, and Dd2^R539T/T38I^ to survive under nutrient deprivation was assessed using growth studies. Parasitaemia of the three lines were assessed by FACS and then diluted down to approximately 0.5% parasitaemia. Lines were then plated in flat-bottomed plates in various nutrient restriction conditions at 5% Hct and incubated in a gassed incubator. Complete media was titrated with a basal salt solution (Additional file 1) to create 100%, 85%, 75%, 65%, 50%, and 20% complete media with and without glucose. Parasitaemia was assessed at 48 h by FACS.

### Ring-stage survival assays to dihydroartemisinin or piperaquine pulse

Ring-stage survival assay (RSA) and piperaquine survival assay (PSA) were performed based on the methodologies described by Kite et al. and Duru et al. respectively [2, 34]. Briefly, Dd2, Dd2^R539T^, and Dd2^R539T/T38I^ parasites were cultivated in 20 mL cultures at 5% hematocrit cultures and synchronized every 46–48 h by a 10 min incubation with 5% sorbitol at 37 °C. After at least two sorbitol synchronizations, 0–3 h ring-stage 1 mL cultures at 2% hematocrit and 1% initial parasitaemia were incubated with either DMSO (Sigma, vehicle), 700 nM DHA (Sigma) in DMSO, 3% methanol (Sigma, vehicle), or 200 nM piperaquine in 3% methanol (kindly provided by Erin Coonahan). DMSO and DHA cultures were washed with 10 mL complete RPMI twice (2500 rpm, 3 min, 9/10 acceleration, 4/10 deacceleration) after 6 h incubation; methanol and piperaquine cultures were washed after 48 h incubation. Thin blood films were prepared from all cultures after a total of 72 h from beginning of each drug or vehicle incubation. Percentage parasite survival was calculated dividing the parasitaemia from a drug-treated culture by the parasitaemia of the culture incubated with the respective drug vehicle, then multiplying the result by 100. To determine parasitaemia, an estimated 10,000 red blood cells from methanol-fixed and 20% Giemsa-stained blood films were counted with oil immersion in a light microscope.

## Results

### A SNP in *atg18* is associated with ART-resistance phenotypes

A GWAS of 782 isolates from Southeast Asia significantly associated a nonsynonymous SNP in *PF3D7_1012900*- (encoding a T38I substitution) with slow parasite clearance rate in patients treated with an ART derivative (p = 5.89E−7) and non-significantly associated with chloroquine resistance (p = 0.0002) (Table 1). This gene putatively encodes *P. falciparum* autophagy-related protein 18 (PfAtg18). Other polymorphisms in autophagy-related genes, putatively encoding Atg7, Atg11, and Atg14, were non-significantly associated with drug resistance phenotypes, but the presence of multiple polymorphisms in the pathway points to parasite modulation of the autophagy pathway as a possible mechanism of drug resistance.

**Table 1:** SNPs in autophagy-related genes from the Tracking Resistance to Artemisinin Collaboration (TRAC) study

Gene	Annotation	Protein	Position	Amino acid change	Mutation type	p value	Phenotype
PF3D7_1343700	Kelch protein K13	Kelch13	1,725,259	X	N	4E−26	Parasite clearance half-life
PF3D7_1012900	Autophagy-related protein 18 (Atg18), putative	Atg18	497,461	T38I	N	5.89E−07	Parasite clearance half-life
PF3D7_1239800	Conserved *Plasmodium* protein, unknown function	Atg11	1,669,294	D2948E	N	3.92E−05	Parasite clearance half-life
PF3D7_1012900	Autophagy-related protein 18 (Atg18), putative	Atg18	497,461	T38I	N	0.0002	Chloroquine IC_50_
PF3D7_1239800	Conserved *Plasmodium* protein, unknown function	Atg11	1,670,910	N3487S	N	0.0002	Parasite clearance half-life
PF3D7_0709400	Cg7 protein	Atg14	426,753	V161E	N	0.0007	Chloroquine IC_50_
PF3D7_1239800	Conserved *Plasmodium* protein, unknown function	Atg11	1,674,565	N4705K	N	0.0007	Piperaquine IC_50_
PF3D7_1126100	ThiF family protein, putative	Atg7	1,018,965	1177C	S	0.0044	Chloroquine IC_50_
PF3D7_1126100	ThiF family protein, putative	Atg7	1,018,965	1177C	S	0.0129	Quinine IC_50_

Proteins listed are putative proteins as annotated by PlasmoDB or through BLAST searches. Kelch13 has been included as a reference, since polymorphisms in this gene have been previously associated with drug resistance phenotypes. The amino acid change for Kelch13 is denoted as “X” to represent several different SNPs that have been found within this gene. Mutation types “N” and “S” indicate non-synonymous and synonymous mutations, respectively. The p values are unadjusted for multiple comparisons. All SNPs that associated with long parasite clearance half-life in the TRAC study were published by Miotto et al. [6]

### PfAtg18 is similar in structure and behaviour to yeast and mammalian homologues

**Fig. 1:**
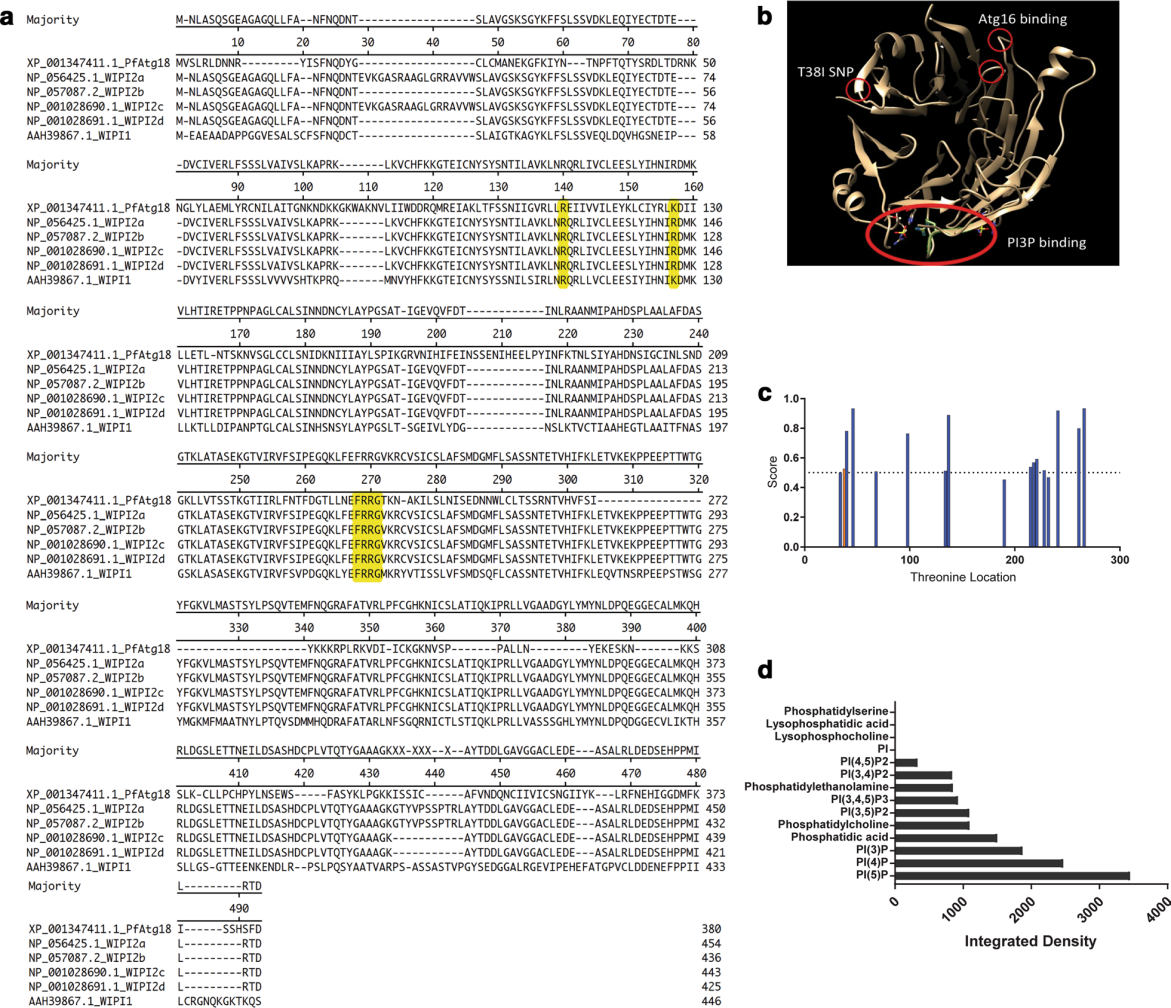
PfAtg18 is capable of binding PI3P. **a** The alignment of the protein sequence of *P. falciparum* PF3D7_1012900, now annotated as autophagy-related gene 18 (atg18), is similar to mammalian homologues, the WD-repeat protein Interacting with Phosphoinositides (WIPI) proteins. Conserved binding domains are highlighted in yellow. WIPI protein FRRG domain binds PI3P while the double arginine residues bind Atg16. **b** Image depicts *P. falciparum* Atg18 protein modeled based on homologous Hsv2 protein in yeast. Putative binding sites for PI3P and Atg16 are highlighted, as well as the location of the T38I SNP (red circles). **c** The threonine amino acids in PfAtg18 are shown with their likelihood of being phosphorylation sites. The T38 site is shown in orange. The threshold (dotted line) is 0.5. **d** Binding of Atg18 from Dd2^R539T^ GFP-tagged Atg18 parasite line to various lipids based on integrated density from an Echelon PIP strip membrane demonstrates binding to PI3P

To understand the role of the mutation in PfAtg18 in drug resistance, its structure and behaviour in *Plasmodium* were investigated further. An alignment of the protein sequence from *PF3D7_1012900* with the homologous WD-repeat protein Interacting with Phosphoinositides (WIPI) proteins confirmed that sequences important for the function of WIPI proteins are also present in PfAtg18 (Fig. 1a). The binding of WIPI to PI3P is one of its principal functions and is facilitated by the FRRG domain. This sequence (highlighted in Fig. 1a) is also present in PfAtg18. WIPI also interacts with Atg16 via its two arginine domains (also highlighted in Fig. 1a). Although PfAtg18 has only one arginine, the function of the second arginine is likely performed by lysine, which is another basic amino acid. A model for PfAtg18 was generated by comparing it structurally to the homologous yeast protein Hsv2, as the human WIPI proteins have not been crystalized. This comparison revealed multiple putative blades on PfAtg18 (Fig. 1b). The PI3P- and Atg16-binding motifs are circled in Fig. 1b, where the FRRG domain is shown to form a pocket, and the arginine/lysine domains are on adjacent blades for protein binding. The SNP of interest was determined to be outside of the binding region for both PI3P and Atg16; however, this does not preclude the possibility that the SNP disrupts binding of PfAtg18 to another protein or lipid. It has been proposed that this area of the homologous human WIPI protein binds to several regulatory proteins [35]. Alternatively, the SNP that mutated threonine to isoleucine (T38I) may impact the function of PfAtg18 through phosphorylation, as a threonine can be phosphorylated and an isoleucine cannot, although protein analysis indicates that the T38 site has a low likelihood of being a phosphorylation site, with a phosphorylation score (0.526) that is just above the threshold (0.500) (Fig. 1c).

Homologous Atg18 proteins bind PI3P as one of the first steps in initiating an autophagy cascade; therefore, in determining possible functions of PfAtg18, it is important to assess if this protein also has these binding capabilities. An Echelon phosphatidylinositol phosphate (PIP) Strip membrane was used to assess binding of PfAtg18 with eight phosphoinositides and seven other lipids. The protein was detected in 10 of the 15 spots, including seven of the eight phosphoinositides (Fig. 1d). Similarly to other Atg18 proteins, PfAtg18 bound PI5P and PI3P at high levels [36].

**Fig. 2:**
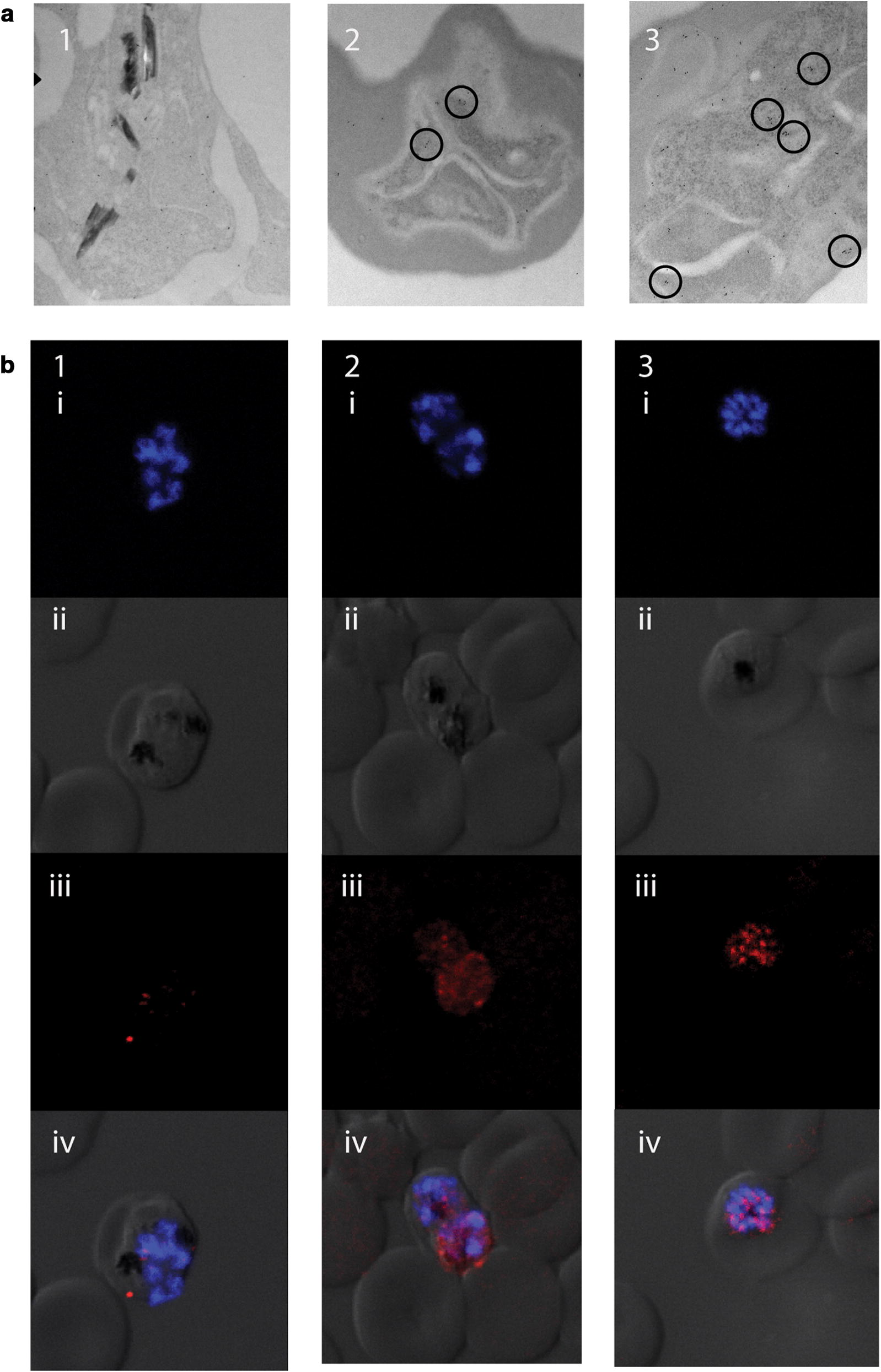
PfAtg18 forms puncta following DHA exposure **a** TEM images of Dd2^R539T^ GFP-Atg18 parasites that were untreated (1) or exposed to 700 nM DHA for 10 min (2) or 1 h (3). Antibodies to GFP were gold-labelled (dots). Labels that congregate to form puncta are highlighted (circles). **b** Late-stage Dd2^R539T^ Atg18-GFP parasites stained with DAPI (blue) and anti-GFP antibody (red). Rows depict images with (i) DAPI only, (ii) DIC only, (iii) anti-GFP only, and (iv) a merged image with all channels. Columns depict parasites that were untreated (1) or exposed to 700 nM DHA for 10 min (2) or 1 h (3)

Homologues of Atg18 are important in the initiation of the autophagy cascade and denote the start of autophagy through the formation of puncta on the nascent autophagosome. (GFP)-Atg18-expressing parasites were generated and transmission electron microscopy (TEM) and confocal microscopy imaging was performed to investigate parasite response to dihydroartemisinin (DHA) as a cellular stressor, known to induce PfAtg8 puncta [37]. Under normal culture conditions, the GFP-Atg18 protein appears diffuse throughout the cytoplasm of late-stage parasites by TEM (Fig. 2A1) and confocal (Fig. 2B1iii) imaging. However, following 700 nM DHA treatment, PfAtg18 proteins start to coalesce within 10 min (Fig. 2A2, B2iii). After 1 h of such treatment, PfAtg18 proteins have formed many puncta, scattered throughout the cytoplasm (Fig. 2A3, B3iii). These results were congruent with previous studies demonstrating rapid puncta formation of WIPI/Atg18 following cellular stressors in mammalian cells [38], thus suggesting that GFP-Atg18 could be used to observe the activation of an autophagy-like pathway in *P. falciparum*. Furthermore, the fact that DHA induces puncta formation of both PfAtg8 and PfAtg18, suggests that DHA may have pro-autophagy effects on parasites.

### An *atg18* SNP is associated with faster growth in nutrient-limited conditions

**Fig. 3:**
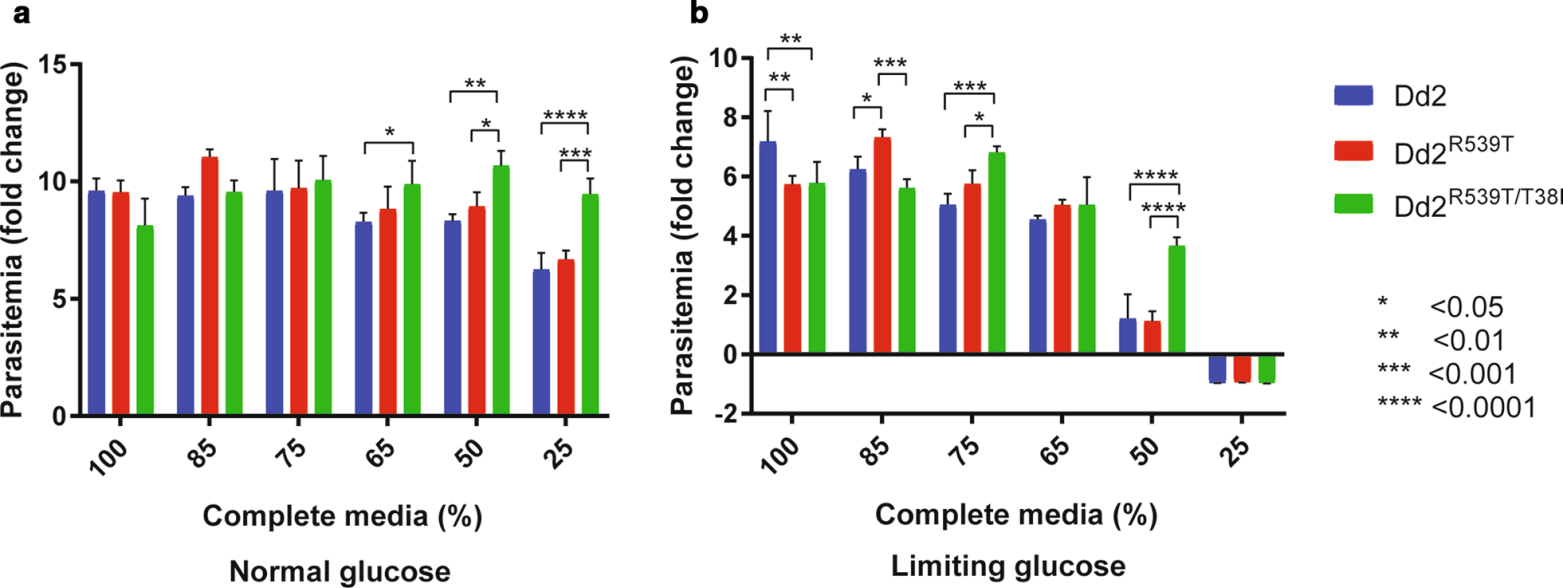
Differential growth rates in nutrient-limited settings Growth over 48 h of isogenic lines Dd2 (blue), Dd2^R539T^ (red), Dd2^R539T/T38I^ (green) in varying nutrient deprivation conditions with glucose at normal (supra-physiologic) media concentration (**a**) and glucose at limiting concentration (**b**)

Given that *atg18* in homologous organisms is important in the autophagy pathway, which is used for nutrient acquisition, the role of PfAtg18 under nutrient-limited conditions was investigated. To determine whether *atg18* T38I, without the variable genetic background found in some contemporary clinical isolates, modulates parasite fitness, the *atg18* T38I locus of Dd2^R539T^ was compared to the *atg18*-edited line, Dd2^R539T/T38I^. The mutation was confirmed by sequencing (Additional file 1). To investigate if the T38I mutation confers a survival advantage to parasites, growth rates of Dd2, Dd2^R539T^, and Dd2^R539T/T38I^ were assessed in a 48-h growth assay with parasitaemia measured by a two-color flow cytometry-based assay using SYBR Green and MitoTracker Deep Red co-staining, as previously described [39]. SYBR Green stains DNA while MitoTracker Deep Red stains intact mitochondria; therefore, double-positive cells represent live parasites. All parasites grew equally well in complete medium (Fig. 3a). A variety of nutrient-limited media were made by titrating complete media with a basal salt solution lacking amino acids, vitamins, and Albumax. Parasites were grown in these nutrient-restricted media and parasitaemia was assessed at 48 h (Fig. 3). In media with 50% reduced nutrients, Dd2^R539T/T38I^ parasites grew significantly faster than their wild-type counterparts (Fig. 3a; vs. Dd2^R539T^, p = 0.02; vs. Dd2, p = 0.002). All parasite lines were still able to grow in 25% complete media; however, Dd2^R539T/T38I^ also outgrew the other lines at this very low concentration of nutrients (vs. Dd2^R539T^, p = 0.0002; vs. Dd2, p < 0.0001) (Fig. 3a). To further understand if the mutation also confers a survival advantage in limiting glucose, the same experiment was performed with and without glucose. When glucose was a limiting nutrient, wild-type parasites struggled to survive in 65% and 50% complete media, and all died in 25% complete media (Fig. 3b). In 50% complete media, Dd2^R539T/T38I^ showed near-normal growth, while Dd2 and Dd2^R539T^ grew significantly slower than Dd2^R539T/T38I^ (both p < 0.0001).

### An *atg18* mutation associates with ex vivo resistance even in the absence of k13 mutations

**Fig. 4:**
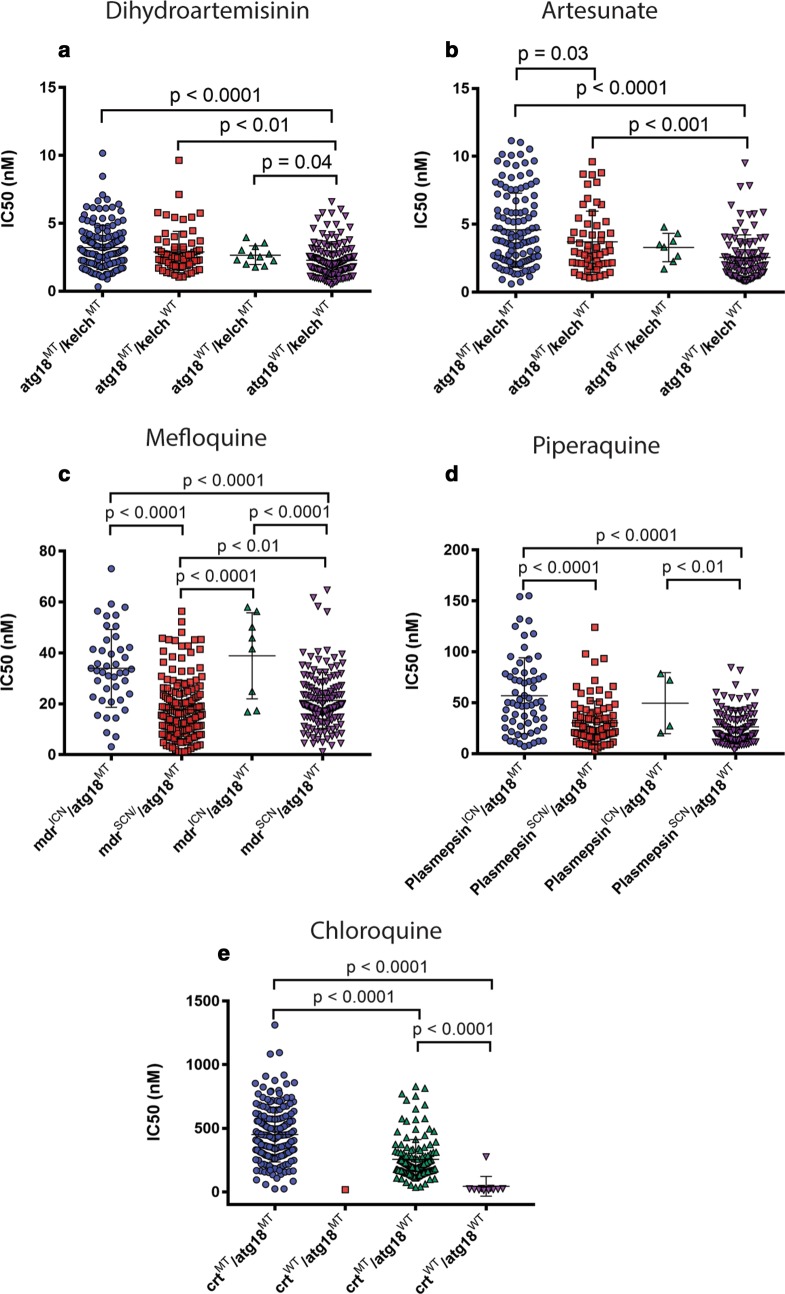
An atg18 T38I SNP is associated with higher IC_50_ values to ART derivatives ex vivo IC_50_ values for six anti-malarial drugs according to the presence of the atg18 T38I polymorphism and/or the polymorphism associated with resistance for that particular drug. **a** DHA and **b** artesunate are synthetic ART derivatives. IC_50_ values for ART derivatives are displayed against any K13-propeller mutation. **c** Mefloquine in the presence or absence of mdr copy number variations. **d** Piperaquine in the presence or absence of plasmepsins 2–3 copy number variations. **e** Chloroquine in the presence or absence of crt mutations. Given the single value in crt^WT^/atg18^MT^, it was not possible to statistically test for differences between this and the other groups. SCN denotes single copy number; ICN denotes increased copy number

Clinical isolates with any *k13*-propeller mutation and the *atg18* T38I mutation had higher ex vivo IC_50_ values for artesunate (t-test, p < 0.0001) and DHA (p < 0.0001) than parasites with wild-type alleles for both *k13*-propeller and *atg18* (Fig. 4a, b). Interestingly, parasites with wild-type *k13*-propeller sequences and the *atg18* T38I mutation had higher IC_50_ values for artesunate (p < 0.001) and DHA (p < 0.01) than parasites with wild-type alleles for both *k13*-propeller and *atg18* (Fig. 4a, b). While this pattern also holds true for chloroquine (t-test, p < 0.0001) and piperaquine (p < 0.0001) when the samples are grouped by *atg18* SNP and *k13* polymorphisms (Additional file 1), the pattern disappears when the samples are grouped by *atg18* SNP and the genetic variant associated with that specific drug’s resistance pattern (*crt* polymorphism for chloroquine or *plasmepsins 2*–*3* copy number for piperaquine). The pattern did not hold true for mefloquine in either case (Fig. 4c–e). These results suggest that the *atg18* T38I mutation may provide additional resistance against ART derivatives, but not partner drugs, even in the absence of *k13* mutations.

### An atg18 mutant parasite line shows differential susceptibility to over 200 compounds

**Fig. 5:**
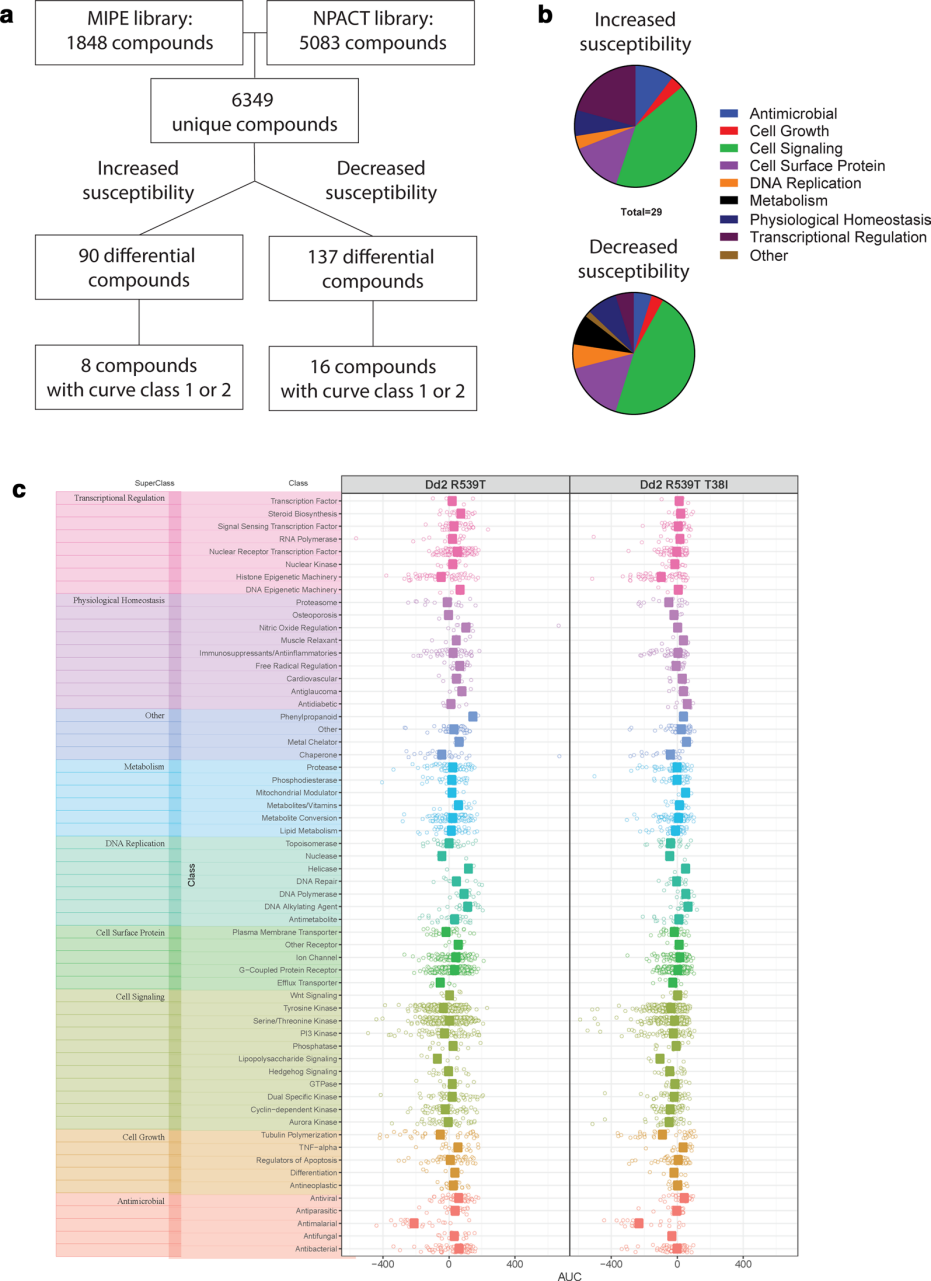
Overview of drug screen against atg18 isogenic lines. **a** NCATS libraries MIPE and NPACT were used to screen 6349 unique compounds. The atg18 T38I SNP was associated with increased susceptibility to 90 compounds. Of these, 8 compounds demonstrated curves in curve classes 1 or 2, demonstrating a shift in susceptibility. On the other hand, this SNP was associated with decreased susceptibility to 137 compounds, 16 of which showed curve classes 1 or 2. **b** The atg18 T38I SNP was associated with increased susceptibility to 90 compounds, 29 of which have known mechanisms of action. The SNP caused decreased susceptibility to 137 compounds, of which 62 were annotated. Drugs are categorized into antimicrobial (blue), cell growth (red), cell signaling (green), cell surface protein (purple), DNA replication (orange), metabolism (black), physiological homeostasis (navy), transcriptional regulation (crimson), or other (tan). **c** AUC values for parent (Dd2^R539T^) and mutant (Dd2^R539T/T38I^) lines organized by superclasses: transcriptional regulation (pink), physiological homeostasis (purple), other (periwinkle blue), metabolism (sky blue), DNA replication (aqua), cell surface protein (green), cell signaling (olive), cell growth (orange), and antimicrobial (coral). These are further broken down into classes by line

The Dd2^R539T^ parent and Dd2^R539T/T38I^ mutant lines were screened against 6349 unique compounds. Of the 6349 unique compounds, 90 were at least 5× more active against Dd2^R539T/T38I^ than Dd2^R539T^ (Fig. 5a). Most of these compounds were not active against Dd2^R539T^ but were active against Dd2^R539T/T38I^; however, eight compounds had a shift in IC_50_ value. Two of these had unconvincing curves and were thus excluded, leaving six compounds that were active against both lines, but more active against the mutant line (Additional file 1). The six compounds to which the mutant was more susceptible were benzethonium chloride, elesclomol, sapitinib, R306465, YM022, and vincristine. A subset of the compounds was annotated (n = 29). Of the 29 differential compounds that were annotated, 41.4% of them acted on cell signaling, 20.7% on transcriptional regulation, 13.8% on a cell surface protein; three compounds were traditional antimicrobials (Fig. 5b). Conversely, 137 compounds were less active against Dd2^R539T/T38I^ than Dd2^R539T^. Sixteen of these had a shift in IC_50_ value (Fig. 5a). Again, a subset of the compounds was annotated (n = 62). Of the 62 differential compounds that were annotated, 46.8% acted on cell signaling, representing statistically significant enrichment (p = 0.006), while 16.1% acted on a cell surface protein, 8.1% on metabolism, 8.1% on physiological homeostasis and again, three compounds were traditional antimicrobials (Fig. 5b). The annotated drug library was organized based on mechanism of action and demonstrated no differences in area under the curve (AUC) between Dd2^R539T^ and Dd2^R539T/T38I^ for any superclass or class of mechanism of action (Fig. 5c). These results indicate that the parent and mutant had differential responses to 227 compounds, and that 24 compounds had a shift in IC_50_ value. Several of these drugs induced ROS or were autophagy modulators, including the metal-chelating drug elesclomol, whose mechanism of action is linked to disruption of oxidative phosphorylation and ROS generation [40, 41].

**Fig. 6:**
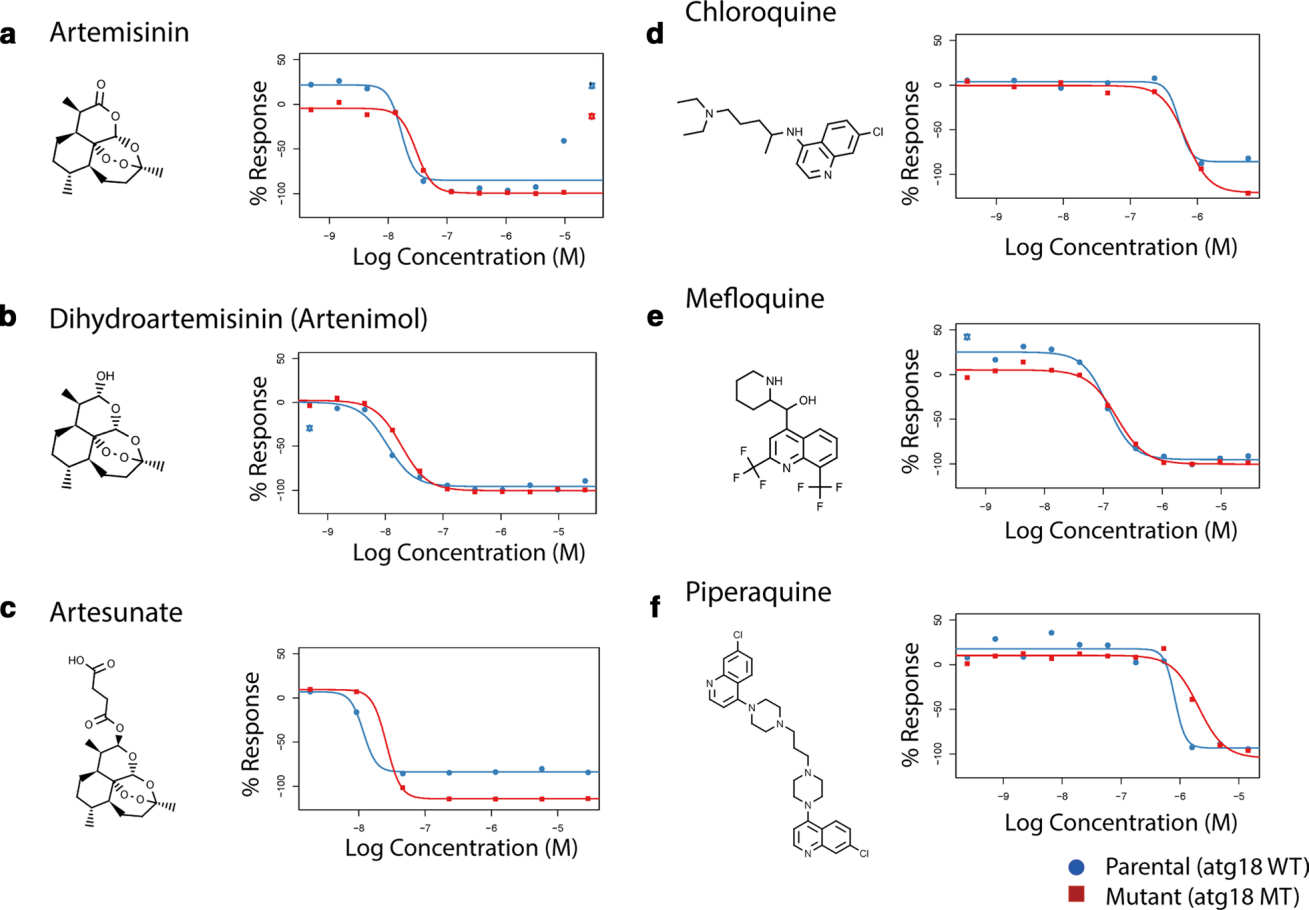
An atg18 SNP does not alter IC_50_ values for common anti-malarial compounds in isogenic lines Dose response curves from **a** ART and two derivatives, **b** DHA, and **c** artesunate, which are used in Cambodia and elsewhere in Southeast Asia. Also shown are dose response curves for **d** chloroquine, **e** mefloquine, and **f** piperaquine, long-lasting partner drugs which have been recently used in Cambodia and elsewhere in Southeast Asia. Parent displayed in blue, mutant in red. Drug structures are also shown

The compound library also included 36 anti-malarial compounds; however, none of their IC_50_ values differed between the parent and mutant lines. Drug response curves of the anti-malarial compounds used in the Greater Mekong Subregion, including ART, two ART derivatives, chloroquine, mefloquine, and piperaquine, are displayed in Fig. 6. Ring-stage survival assay (RSA) and piperaquine survival assay (PSA) did not show a difference in survival in parent or mutant lines, though both Dd2^R539T^ and Dd2^R539T/T38I^ showed increased survival above Dd2 in the RSA (p = 0.02 and p = 0.04, respectively) and PSA (p = 0.04 and p = 0.03, respectively) (Additional file 1). These findings suggest that either this SNP does not directly impact parasite response to these anti-malarials or that a change in IC_50_ value is not suitable to detect the impact of the *atg18* T38I SNP on resistance.

## Discussion

The proposed mechanisms of ART resistance suggest that an autophagy-like mechanism could play a role in modulating resistance in *P. falciparum*. Several SNPs in autophagy-related proteins were associated with drug resistance phenotypes; however, this study chose to focus on *atg18* based on the level of significance of its SNP in the GWAS. A second report associated a similar SNP in *atg18* with ART derivative and piperaquine resistance phenotypes in an independent cohort in the border region of China and Myanmar, confirming the potential relevance of this SNP in conferring anti-malarial resistance [42].

PfAtg18 is likely a homologue of WIPI/Atg18 with a similar binding capacity and behaviour pattern following stress. The ability of PfAtg18 to bind to PI3P is significant. Homologous Atg18/WIPI proteins also bind PI3P and, therefore, the conservation of binding suggests potential preservation of part of the autophagy pathway. Further, PI3P been implicated in drug resistance in *P. falciparum* from work by Mbengue and colleagues [18], who suggested there is a relationship between PI3P and artemisinin resistance. While autophagy-related proteins are involved in apicoplast inheritance [13], the results of this study indicate that autophagy-related proteins may have alternative functions outside of the apicoplast, as the only apparently essential function of the apicoplast is to provide isopentyl pyrophosphate (IPP) [43].

If PfAtg18 is involved in an autophagy-like pathway in *Plasmodium*, this SNP may modulate its activity. A number of mutations in homologous WIPI proteins have been described in various cancers [44]. The diversity of mutations found in WIPI proteins in cancer suggests that these mutations provide a survival advantage. For a mutation to be advantageous in cancer, it must facilitate cancer cell growth, for example through nutrient acquisition, dealing with cellular stress or several other pro-survival mechanisms [45, 46]. Thus, the plasmodial *atg18* T38I mutation may similarly offer a survival benefit. Given the ability of the Dd2^R539T/T38I^ mutant to survive better in nutrient-limited settings than either its parent Dd2^R539T^ line or the original Dd2 line, the T38I SNP may therefore represent a fitness advantage by growing more effectively in the nutrient-limited setting of parasite infection and acute malaria. This alone or in addition to a drug-resistance phenotype, could select for the T38I SNP. Despite allowing for increased survival of early ring-stage parasites, *k13* polymorphisms have been shown to cause growth deficits in vitro [47]. The T38I mutation could, therefore, compensate for the loss of fitness caused by *k13* or other unknown polymorphisms in the parasite population. DHA-resistant isolates have been shown to grow more rapidly in stressful conditions, as evidenced by a decreased growth rate for sensitive parasites at high parasitaemia [48]. At higher parasitaemia, growth media, which is normally supra-physiologic, may become rapidly depleted, thus creating a nutrient-limited media. Therefore, these results align with the finding that efficient growth in nutrient deprivation may be driving selection. Further, these stressful, oxidative stress-inducing conditions would typically slow parasite growth [49]. One possible explanation is that if a parasite is better equipped to handle damage by oxidative stress, perhaps by employing autophagy-like mechanisms, that parasite would likely outcompete other parasites in a population.

Growth of *Plasmodium* parasites appears to be susceptible to changes in the immediate host environment. Dietary restrictions in mice have been shown to be protective against cerebral malaria by *Plasmodium berghei* [50, 51]. Refeeding of patients with *P. falciparum* in the hospital setting has been associated with an increase in parasitaemia [52]. In culture supplemented with sera from calorie-restricted mice as compared to fed mice, parasites formed fewer merozoites during schizogony, thus reducing overall parasitaemia [53]. Thus, parasitaemia is intricately tied to host nutrition status. The serine/threonine kinase KIN, which is putatively homologous to AMP-activated kinase (AMPK), has been implicated in nutrient sensing in *Plasmodium* in the absence of TOR proteins [53]. AMPK is another regulator of autophagy along with mTOR [54]. An autophagy-like pathway may therefore play a role in nutrient sensing in *Plasmodium* and may modulate parasite virulence based on the ability of the parasite to obtain limited nutrients.

Many anti-malarial drugs target metabolic pathways [55]. An improvement in a parasite’s ability to survive low nutrient conditions not only benefits the parasite in terms of its growth rate, but also enables a parasite to evade drugs that may target various metabolic pathways. While a drug may block one metabolic pathway, another pathway that was previously redundant may become essential. The ability to grow more efficiently in nutrient-limited media suggests changes in the metabolism of parasites carrying this mutation, which could allow the parasite to survive drug treatment. Again, the ability to survive a drug pulse based on the parasite’s ability to employ alternate metabolic pathways may appear inconsequential in a drug screen in complete media, where nutrient limitation is not a factor. Experiments examining parasite responses to drugs in a variety of media are ongoing.

The drug screen revealed that the mutant line is differentially susceptible to > 200 compounds, including a number of compounds that would likely modulate autophagy, including vincristine [56], sapitinib [57, 58], and gefitinib [59, 60], whereas others modulate ROS, including elesclomol [41, 61], olmesartan [62], and paeoniflorin [63]. Despite these effects on the parasite’s susceptibility to other compounds, the *atg18* T38I SNP did not change IC_50_ values for the tested anti-malarial compounds. This was surprising given the results of the ex vivo IC_50_ experiments and GWAS. It is possible that, like *k13* polymorphisms, the contribution of the *atg18* polymorphism to resistance cannot be detected using a 72-h assay [64, 65]. Thus, studies are underway to determine if this SNP modulates several other phenotypes including those measured by dormancy assays. Additionally, the effects of the SNP may be important only in the context of other background mutations, which are absent in the Dd2^R539T^ line.

It is possible that the T38I SNP changes the function of PfAtg18 to promote more efficient nutrient acquisition through an autophagy-like pathway. This could allow for either increased fitness leading to expansion within the parasite population or the ability of a parasite to survive drug treatment by utilizing its autophagy-like pathway to survive in the absence of a blocked metabolic pathway. Further studies, including a more detailed characterization of the function of PfAtg18, are necessary to determine if this is indeed the case.

## Additional file

**Additional file 1.** Nutrient deprivation media compositions, ex vivo IC_50_ values in parasites with Kelch13 mutations, dose response curves for the drug screen compounds with differential susceptibility, and RSA and PSA results.

## Abbreviations

AMPK: AMP-activated kinase
*arps10*: *apicoplast ribosomal protein S10*
AUC: area under the curve
ART: artemisinin
ACT: artemisinin-based combination therapy
*atg18*: *autophagy*-*related gene 18*
PfAtg8: autophagy-related protein 8
BSD: blasticidin
*crt*: *chloroquine resistance transporter*
DAPI: 4′,6-diamidino-2-phenylindole
*fd*: *ferrodoxin*
GWAS: genome-wide association study
GFP: green fluorescent protein
IC_50_: half-maximal inhibitory concentration
hDHFR: human dihydrofolate reductase
IPP: isopentyl pyrophosphate
*k13*: *kelch13*
MaxR: maximum dose response
MIPE: Mechanism Interrogation Plate
NCATS: National Center for Advancing Translational Sciences
*mdr2*: *multidrug*-*resistance protein 2*
PfAtg18: *P. falciparum* autophagy-related protein 18
PIP: phosphatidylinositol phosphate
PI3P: phosphatidylinositol 3-phosphate
PAM: protospacer adjacent motif
sgRNA: single guide RNA
SNPs: single nucleotide polymorphisms
TRAC: Tracking Resistance to Artemisinin Collaboration
TEM: transmission electron microscopy
UPR: unfolded protein response
WIPI: WD-repeat protein Interacting with Phosphoinositides
WGS: whole genome sequencing
yDHODH: yeast dihydroorotate dehydrogenase

## References

[1] Amaratunga C, Lim P, Suon S, Sreng S, Mao S, Sopha C (2016). Dihydroartemisinin-piperaquine resistance in *Plasmodium falciparum* malaria in Cambodia: a multisite prospective cohort study. Lancet Infect Dis..

[2] Duru V, Khim N, Leang R, Kim S, Domergue A, Kloeung N (2015). *Plasmodium falciparum* dihydroartemisinin-piperaquine failures in Cambodia are associated with mutant K13 parasites presenting high survival rates in novel piperaquine in vitro assays: retrospective and prospective investigations. BMC Med..

[3] Ashley EA, Dhorda M, Fairhurst RM, Amaratunga C, Lim P, Suon S (2014). Spread of artemisinin resistance in *Plasmodium falciparum* malaria. N Engl J Med.

[4] Ghorbal M, Gorman M, Macpherson CR, Martins RM, Scherf A, Lopez-Rubio JJ (2014). Genome editing in the human malaria parasite *Plasmodium falciparum* using the CRISPR-Cas9 system. Nat Biotechnol.

[5] Straimer J, Gnadig NF, Witkowski B, Amaratunga C, Duru V, Ramadani AP (2015). Drug resistance. K13-propeller mutations confer artemisinin resistance in *Plasmodium falciparum* clinical isolates. Science.

[6] Miotto O, Amato R, Ashley EA, MacInnis B, Almagro-Garcia J, Amaratunga C (2015). Genetic architecture of artemisinin-resistant *Plasmodium falciparum*. Nat Genet.

[7] Taylor SM, Parobek CM, DeConti DK, Kayentao K, Coulibaly SO, Greenwood BM (2015). Absence of putative artemisinin resistance mutations among *Plasmodium falciparum* in Sub-Saharan Africa: a molecular epidemiologic study. J Infect Dis.

[8] Mukherjee A, Bopp S, Magistrado P, Wong W, Daniels R, Demas A (2017). Artemisinin resistance without pfkelch13 mutations in *Plasmodium falciparum* isolates from Cambodia. Malar J..

[9] Kheang ST, Sovannaroth S, Ek S, Chy S, Chhun P, Mao S (2017). Prevalence of K13 mutation and Day-3 positive parasitaemia in artemisinin-resistant malaria endemic area of Cambodia: a cross-sectional study. Malar J..

[10] Ouattara A, Kone A, Adams M, Fofana B, Maiga AW, Hampton S (2015). Polymorphisms in the K13-propeller gene in artemisinin-susceptible *Plasmodium falciparum* parasites from Bougoula-Hameau and Bandiagara, Mali. Am J Trop Med Hyg..

[11] Muwanguzi J, Henriques G, Sawa P, Bousema T, Sutherland CJ, Beshir KB (2016). Lack of K13 mutations in *Plasmodium falciparum* persisting after artemisinin combination therapy treatment of Kenyan children. Malar J..

[12] Cooper RA, Conrad MD, Watson QD, Huezo SJ, Ninsiima H, Tumwebaze P (2015). Lack of artemisinin resistance in *Plasmodium falciparum* in Uganda based on parasitological and molecular assays. Antimicrob Agents Chemother.

[13] Bansal P, Tripathi A, Thakur V, Mohmmed A, Sharma P (2017). Autophagy-related protein ATG18 regulates apicoplast biogenesis in Apicomplexan parasites. MBio..

[14] Klionsky DJ, Abdelmohsen K, Abe A, Abedin MJ, Abeliovich H, Acevedo Arozena A, et al. Guidelines for the use and interpretation of assays for monitoring autophagy 3rd ed. Autophagy. 2016;12:1–222. 10.1080/15548627.2015.1100356 PMC4835977 26799652

[15] Dogovski C, Xie SC, Burgio G, Bridgford J, Mok S, McCaw JM (2015). Targeting the cell stress response of *Plasmodium falciparum* to overcome artemisinin resistance. PLoS Biol.

[16] Li J, Zhou B (2010). Biological actions of artemisinin: insights from medicinal chemistry studies. Molecules.

[17] Thorpe GW, Fong CS, Alic N, Higgins VJ, Dawes IW (2004). Cells have distinct mechanisms to maintain protection against different reactive oxygen species: oxidative-stress-response genes. Proc Natl Acad Sci USA.

[18] Mbengue A, Bhattacharjee S, Pandharkar T, Liu H, Estiu G, Stahelin RV (2015). A molecular mechanism of artemisinin resistance in *Plasmodium falciparum* malaria. Nature.

[19] Mok S, Ashley EA, Ferreira PE, Zhu L, Lin Z, Yeo T (2015). Population transcriptomics of human malaria parasites reveals the mechanism of artemisinin resistance. Science.

[20] Behrends C, Sowa ME, Gygi SP, Harper JW (2010). Network organization of the human autophagy system. Nature.

[21] Lim P, Dek D, Try V, Eastman RT, Chy S, Sreng S (2013). Ex vivo susceptibility of *Plasmodium falciparum* to antimalarial drugs in western, northern, and eastern Cambodia, 2011-2012: association with molecular markers. Antimicrob Agents Chemother.

[22] Bacon DJ, Latour C, Lucas C, Colina O, Ringwald P, Picot S (2007). Comparison of a SYBR green I-based assay with a histidine-rich protein II enzyme-linked immunosorbent assay for in vitro antimalarial drug efficacy testing and application to clinical isolates. Antimicrob Agents Chemother.

[23] Wu Y, Sifri CD, Lei HH, Su XZ, Wellems TE (1995). Transfection of *Plasmodium falciparum* within human red blood cells. Proc Natl Acad Sci USA.

[24] Deitsch KW, Driskill CL, Wellems TE (2001). Transformation of malaria parasites by the spontaneous uptake and expression of DNA from human erythrocytes. Nucleic Acids Res.

[25] Fidock DA, Wellems TE (1997). Transformation with human dihydrofolate reductase renders malaria parasites insensitive to WR99210 but does not affect the intrinsic activity of proguanil. Proc Natl Acad Sci USA.

[26] Mathews Griner LA, Guha R, Shinn P, Young RM, Keller JM, Liu D (2014). High-throughput combinatorial screening identifies drugs that cooperate with ibrutinib to kill activated B-cell-like diffuse large B-cell lymphoma cells. Proc Natl Acad Sci USA.

[27] Inglese J, Auld DS, Jadhav A, Johnson RL, Simeonov A, Yasgar A (2006). Quantitative high-throughput screening: a titration-based approach that efficiently identifies biological activities in large chemical libraries. Proc Natl Acad Sci USA.

[28] Yuan J, Johnson RL, Huang R, Wichterman J, Jiang H, Hayton K (2009). Genetic mapping of targets mediating differential chemical phenotypes in *Plasmodium falciparum*. Nat Chem Biol.

[29] Smilkstein M, Sriwilaijaroen N, Kelly JX, Wilairat P, Riscoe M (2004). Simple and inexpensive fluorescence-based technique for high-throughput antimalarial drug screening. Antimicrob Agents Chemother.

[30] Bennett TN, Paguio M, Gligorijevic B, Seudieu C, Kosar AD, Davidson E (2004). Novel, rapid, and inexpensive cell-based quantification of antimalarial drug efficacy. Antimicrob Agents Chemother.

[31] Wang Y, Jadhav A, Southal N, Huang R, Nguyen D-T (2010). A grid algorithm for high throughput fitting of dose-response curve data. Curr Chem Genomics..

[32] Blom N, Gammeltoft S, Brunak S (1999). Sequence and structure-based prediction of eukaryotic protein phosphorylation sites. J Mol Biol.

[33] Faris R, Moore RA, Ward A, Race B, Dorward DW, Hollister JR (2017). Cellular prion protein is present in mitochondria of healthy mice. Sci Rep..

[34] Kite WA, Melendez-Muniz VA, Moraes Barros RR, Wellems TE, Sa JM (2016). Alternative methods for the *Plasmodium falciparum* artemisinin ring-stage survival assay with increased simplicity and parasite stage-specificity. Malar J..

[35] Proikas-Cezanne T, Waddell S, Gaugel A, Frickey T, Lupas A, Nordheim A (2004). WIPI-1alpha (WIPI49), a member of the novel 7-bladed WIPI protein family, is aberrantly expressed in human cancer and is linked to starvation-induced autophagy. Oncogene.

[36] Vicinanza M, Korolchuk Viktor I, Ashkenazi A, Puri C, Menzies Fiona M, Clarke Jonathan H (2015). PI(5)P regulates autophagosome biogenesis. Mol Cell.

[37] Mott BT, Eastman RT, Guha R, Sherlach KS, Siriwardana A, Shinn P (2015). High-throughput matrix screening identifies synergistic and antagonistic antimalarial drug combinations. Sci Rep..

[38] Proikas-Cezanne T, Ruckerbauer S, Stierhof YD, Berg C, Nordheim A (2007). Human WIPI-1 puncta-formation: a novel assay to assess mammalian autophagy. FEBS Lett.

[39] Amaratunga C, Neal AT, Fairhurst RM (2014). Flow cytometry-based analysis of artemisinin-resistant *Plasmodium falciparum* in the ring-stage survival assay. Antimicrob Agents Chemother.

[40] Blackman RK, Cheung-Ong K, Gebbia M, Proia DA, He S, Kepros J (2012). Mitochondrial electron transport is the cellular target of the oncology drug elesclomol. PLoS ONE.

[41] Kirshner JR, He S, Balasubramanyam V, Kepros J, Yang CY, Zhang M (2008). Elesclomol induces cancer cell apoptosis through oxidative stress. Mol Cancer Ther.

[42] Wang Z, Cabrera M, Yang J, Yuan L, Gupta B, Liang X (2016). Genome-wide association analysis identifies genetic loci associated with resistance to multiple antimalarials in *Plasmodium falciparum* from China-Myanmar border. Sci Rep..

[43] Yeh E, DeRisi JL (2011). Chemical rescue of malaria parasites lacking an apicoplast defines organelle function in blood-stage *Plasmodium falciparum*. PLoS Biol.

[44] Proikas-Cezanne T, Takacs Z, Dönnes P, Kohlbacher O (2015). WIPI proteins: essential PtdIns3P effectors at the nascent autophagosome. J Cell Sci.

[45] Hanahan D, Weinberg RA (2000). The hallmarks of cancer. Cell.

[46] Hanahan D, Weinberg RA (2011). Hallmarks of cancer: the next generation. Cell.

[47] Straimer J, Gnädig NF, Stokes BH, Ehrenberger M, Crane AA, Fidock DA (2017). *Plasmodium falciparum* k13 mutations differentially impact ozonide susceptibility and parasite fitness in vitro. MBio..

[48] Cui L, Wang Z, Miao J, Miao M, Chandra R, Jiang H (2012). Mechanisms of in vitro resistance to dihydroartemisinin in *Plasmodium falciparum*. Mol Microbiol.

[49] Becker K, Tilley L, Vennerstrom JL, Roberts D, Rogerson S, Ginsburg H (2004). Oxidative stress in malaria parasite-infected erythrocytes: host–parasite interactions. Int J Parasitol.

[50] Mejia P, Trevino-Villarreal JH, Hine C, Harputlugil E, Lang S, Calay E (2015). Dietary restriction protects against experimental cerebral malaria via leptin modulation and T-cell mTORC1 suppression. Nat Commun..

[51] Hunt NH, Manduci N, Thumwood CM (1993). Amelioration of murine cerebral malaria by dietary restriction. Parasitology.

[52] Murray MJ, Murray NJ, Murray AB, Murray MB (1975). Refeeding-malaria and hyperferraemia. Lancet.

[53] Mancio-Silva L, Slavic K, Grilo Ruivo MT, Grosso AR, Modrzynska KK, Vera IM (2017). Nutrient sensing modulates malaria parasite virulence. Nature.

[54] Kim J, Kundu M, Viollet B, Guan K-L (2011). AMPK and mTOR regulate autophagy through direct phosphorylation of Ulk1. Nat Cell Biol.

[55] Delves M, Plouffe D, Scheurer C, Meister S, Wittlin S, Winzeler EA (2012). The activities of current antimalarial drugs on the life cycle stages of *Plasmodium*: a comparative study with human and rodent parasites. PLoS Med..

[56] Groth-Pedersen L, Ostenfeld MS, Høyer-Hansen M, Nylandsted J, Jäättelä M (2007). Vincristine induces dramatic lysosomal changes and sensitizes cancer cells to lysosome-destabilizing Siramesine. Cancer Res.

[57] Morrison G, Fu X, Shea M, Nanda S, Giuliano M, Wang T (2014). Therapeutic potential of the dual EGFR/HER2 inhibitor AZD8931 in circumventing endocrine resistance. Breast Cancer Res Treat.

[58] Lum JJ, Bauer DE, Kong M, Harris MH, Li C, Lindsten T (2005). Growth factor regulation of autophagy and cell survival in the absence of apoptosis. Cell.

[59] Tan X, Thapa N, Sun Y, Anderson RA (2015). A kinase-independent role for EGF receptor in autophagy initiation. Cell.

[60] Liu JT, Li WC, Gao S, Wang F, Li XQ, Yu HQ (2015). Autophagy inhibition overcomes the antagonistic effect between gefitinib and cisplatin in epidermal growth factor receptor mutant non–small-cell lung cancer cells. Clin Lung Cancer..

[61] Hambright HG, Ghosh R (2017). Autophagy: in the cROSshairs of cancer. Biochem Pharmacol.

[62] Yu KY, Wang YP, Wang LH, Jian Y, Zhao XD, Chen JW (2014). Mitochondrial KATP channel involvement in angiotensin II-induced autophagy in vascular smooth muscle cells. Basic Res Cardiol.

[63] Zhu L, Wei W, Zheng YQ, Jia XY (2005). Effects and mechanisms of total glucosides of paeony on joint damage in rat collagen-induced arthritis. Inflamm Res.

[64] Witkowski B, Amaratunga C, Khim N, Sreng S, Chim P, Kim S (2013). Novel phenotypic assays for the detection of artemisinin-resistant *Plasmodium falciparum* malaria in Cambodia: in vitro and ex vivo drug-response studies. Lancet Infect Dis..

[65] Witkowski B, Khim N, Chim P, Kim S, Ke S, Kloeung N (2013). Reduced artemisinin susceptibility of *Plasmodium falciparum* ring stages in western Cambodia. Antimicrob Agents Chemother.

