# Supplementary material for: A single nucleotide polymorphism in the Plasmodium falciparum atg18 gene associates with artemisinin resistance and confers enhanced parasite survival under nutrient deprivation
Source: Malar J. 2018 Oct 26;17:391. doi: 10.1186/s12936-018-2532-x (PMC6204056; doi:10.1186/s12936-018-2532-x)
Supplement: Supplementary file 1 — Additional file 1. Nutrient deprivation media compositions, ex vivo IC50 values in parasites with Kelch13 mutations, dose response curves for the drug screen compounds with differential susceptibility, and RSA and PSA results. [file 12936_2018_2532_MOESM1_ESM.pdf]

Supplemental Data:

Nutrient deprivation media compositions

**Reagent**	**Complete**	**Basal salt solution with glucose**	**Basal salt solution**
10x Inorganic Salts*	50 mL	50 mL	50 mL
10x Na_2HPO_4 **	50 mL	50 mL	50 mL
$(Na)HCO_3$	1.125 g	1.125 g	1.125 g
Glucose	2 g	2 g	------
100x RPMI 1640 Vitamins	5 mL	------	------
Glutathione (reduced) (1mg/mL)	500 µL	500 µL	500 µL
Hepes	3 g	3 g	3 g
Phenol Red (optional)	2.5 mg	2.5 mg	2.5 mg
Hypoxanthine	25 mg	25 mg	25 mg
Gentamicin	100 µL	100 µL	100 µL
50x RPMI 1640 Amino Acid Solution without L-Glutathione	10 mL	------	------
L-Glutamine	300 mg	------	------
Albumax II	2.5 g	------	------
ddH_2O	to 500 mL final volume	to 500 mL final volume	to 500 mL final volume

*Recipe as used in Fennell et al. 2009 [1]. *10X Inorganic salts mix (1 L): 1 g Ca(NO3)2•4H2O, 4 g KCl, 0.5 g MgSO4 (anhydrous), 60 g NaCl, ddH2O to 1 L final volume; **10x Na_2HPO (1L): 8 g Na_2HPO, ddH2O to 1 L final volume.*

Ex-vivo IC_{50} values in parasites with Kelch13 mutations

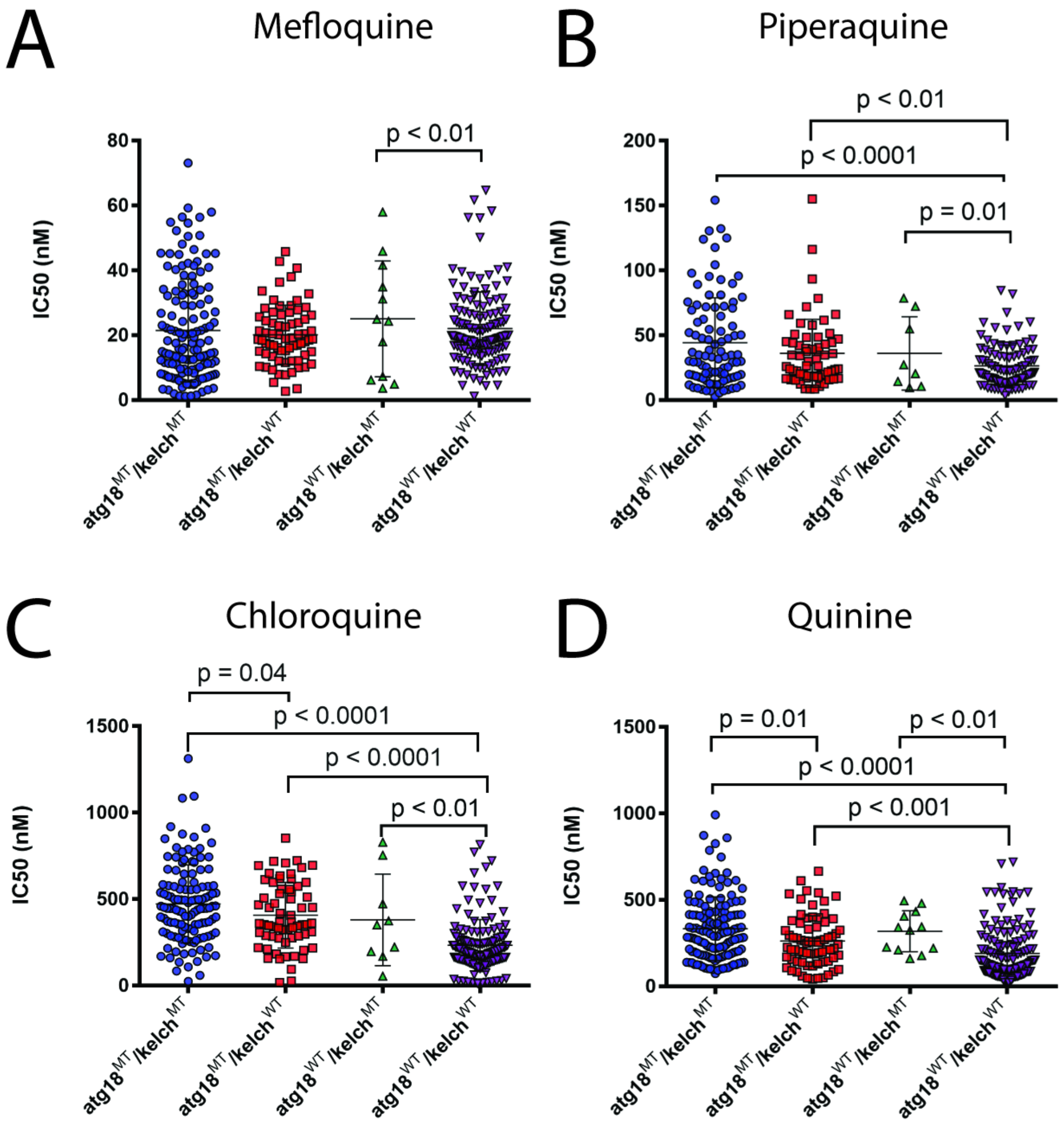

IC_{50} values for four antimalarial drugs according to the presence of any K13-propeller mutation and the atg18 T38I mutation in Cambodian parasite isolates. Mefloquine (A), piperaquine (B), and chloroquine (C) are drugs that are currently or have been previously used as monotherapy or as ACT partner drugs. Quinine (D) is not routinely used in Cambodia.

Drug screen compounds with differential susceptibility favoring parent

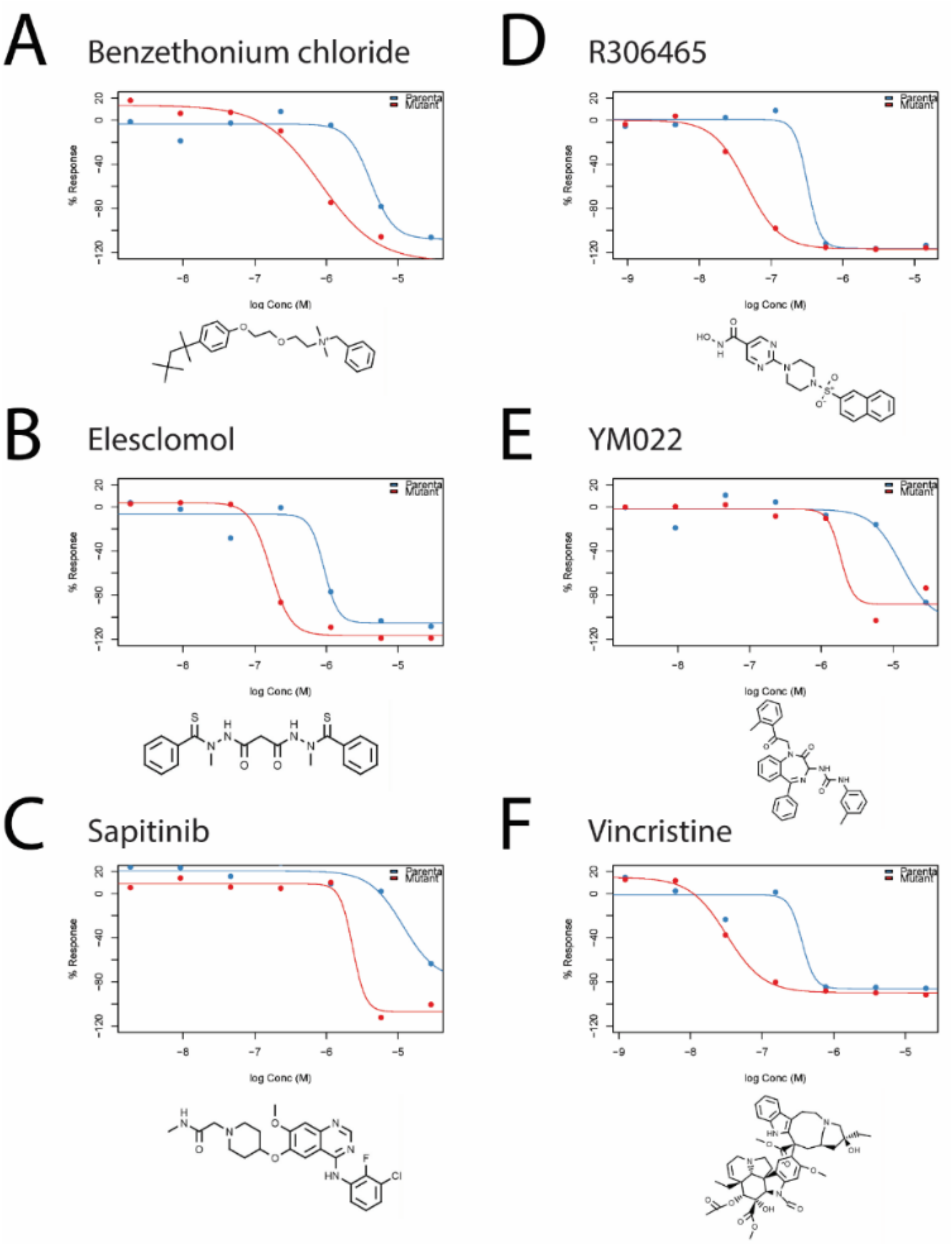

Dose response curves for (A) benzethonium chloride, (B) elesclomol, (C) sapitinib, (D) R306465, (E) YM022, and (F) vincristine. Parent displayed in blue, mutant in red. Drug structures are also shown.

Drug screen compounds with differential susceptibility favoring mutant

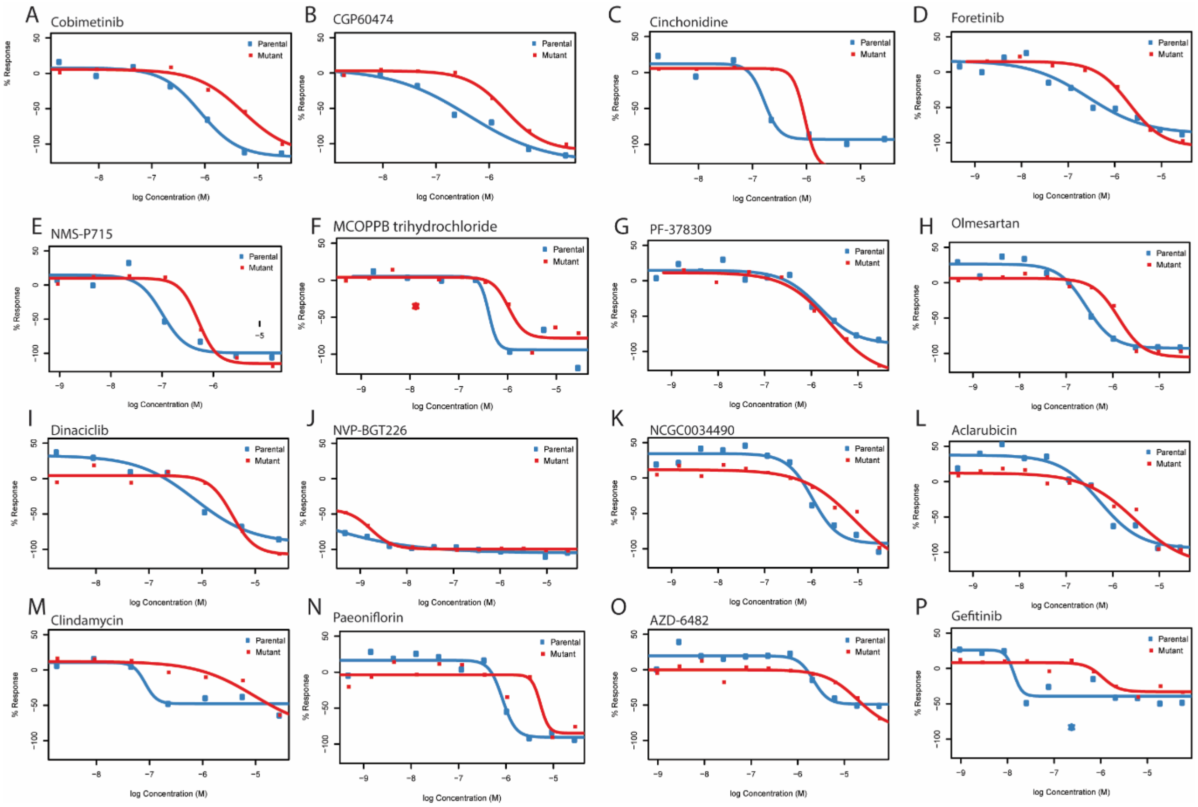

Dose response curves for (A) cobimetinib, (B) CGP60474, (C) cinchonidine, (D) foretinib, (E) NMS-P715, (F) MCOPPB trihydrochloride, (G) PF-378309, (H) olmesartan, (I) dinaciclib, (J) NVP-BGT226, (K) NCGC0034490, (L) aclarubicin, (M) clindamycin, (N) paeoniflorin, (O) AZD-6482, and (P) gefitinib. Parent displayed in blue, mutant in red.

T38I SNP does not modulate DHA or piperaquine sensitivity in specific ring-stage assays

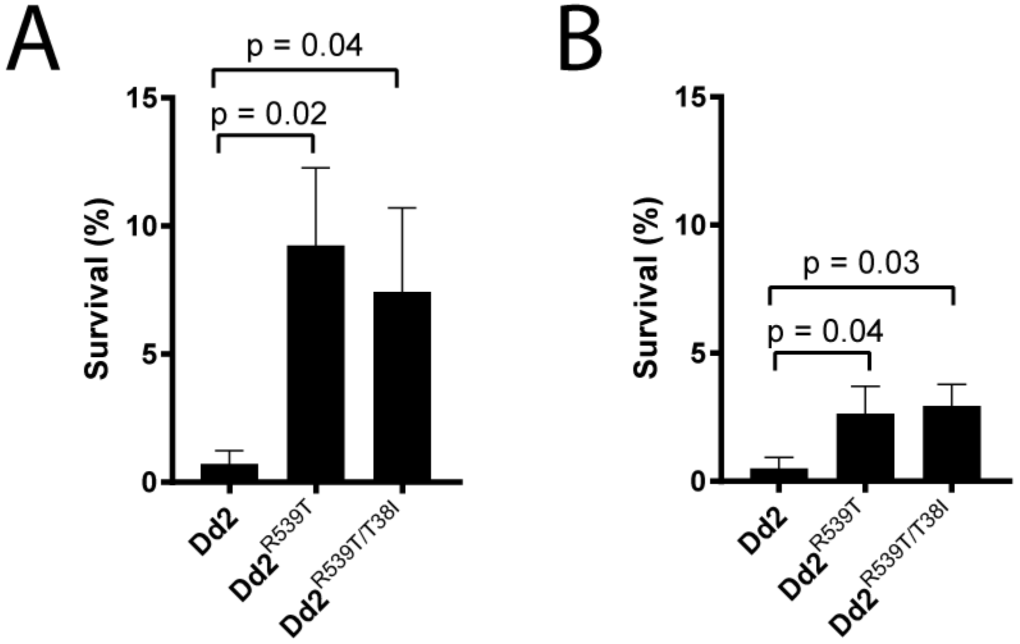

Ring-stage parasites from Dd2, Dd2^{R539T}, and Dd2$^{R539T/T38I}$ were isolated and subjected to drug pulses of DHA (A) or piperaquine (B) as per previously described RSA and PSA assays respectively.

Confirmation of CRISPR editing

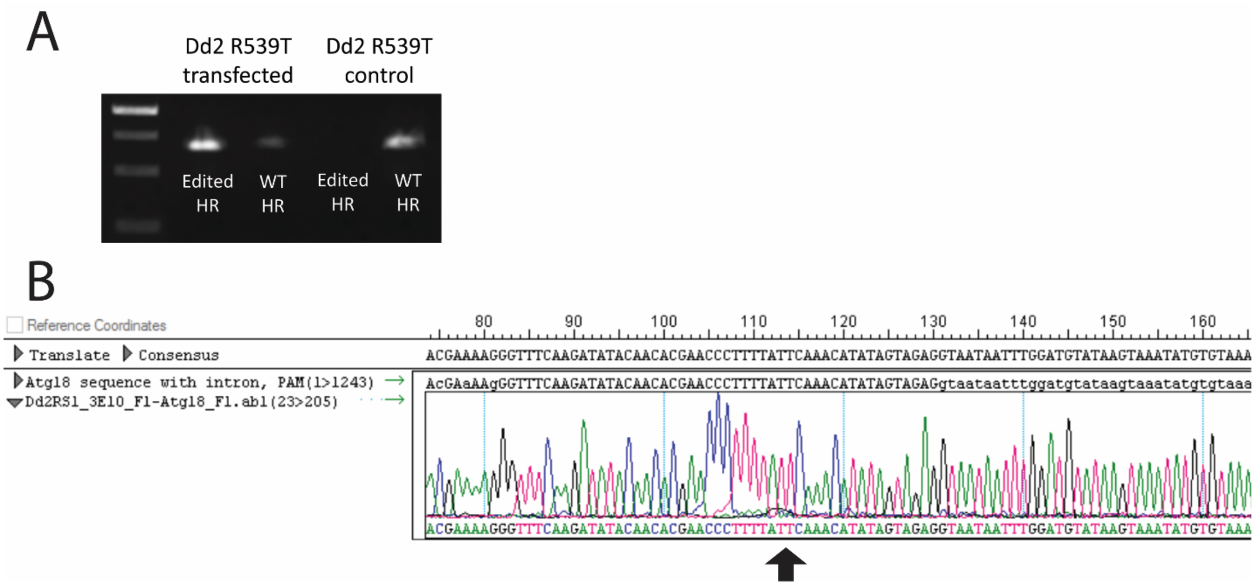

(A) Parasites that grew following drug pressure were screened by PCR to determine if they had the edited homology region. The representative gel here shows a heterogeneous population of parasites with and without editing, as there are bands corresponding to reactions using primers specific for the edited (column 1) and non-edited (column 2) homology regions. The controls showed no editing (column 3). (B) Shown is a representative image of a chromatogram demonstrating editing of the locus of interest (T to I). The arrow denotes the changed base pair causing the sequence to encode isoleucine.

Supplemental Citation

1. Fennell C, Babbitt S, Russo I, Wilkes J, Ranford-Cartwright L, Goldberg DE, Doerig C. **PfeIK1, a eukaryotic initiation factor 2alpha kinase of the human malaria parasite *Plasmodium falciparum*, regulates stress-response to amino-acid starvation.** *Malaria Journal* 2009, **8:**99.
